# *Aire* and *Fezf2* Shape the Medullary Thymic Epithelial Cell Immunopeptidome for Central Tolerance

**DOI:** 10.1111/imm.70176

**Published:** 2026-07-23

**Authors:** Geraldo Aleixo Passos, Vitor Marcel Faça, Eduardo Antonio Donadi, Ana Paula Masson, Carlos Crippa Coletto, Gustavo Ronconi Roza

**Affiliations:** ^1^ Laboratory of Genetics and Molecular Biology, Department of Basic and Oral Biology, Ribeirão Preto School of Dentistry University of São Paulo Ribeirão Preto Brazil; ^2^ Molecular Immunogenetics Group, Department of Genetics, Ribeirão Preto Medical School University of São Paulo Ribeirão Preto Brazil; ^3^ Laboratory of Cancer Proteomics, Department of Biochemistry and Immunology, Ribeirão Preto Medical School University of São Paulo Ribeirão Preto Brazil; ^4^ Division of Clinical Immunology, Department of Medicine, Ribeirão Preto Medical School University of São Paulo Ribeirão Preto Brazil

**Keywords:** *Aire*, central tolerance, *Fezf2*, immunopeptidome, mTEC, promiscuous gene expression

## Abstract

Medullary thymic epithelial cells (mTECs) are central to immune self‐tolerance owing to their capacity to express and present a diverse repertoire of self‐derived peptides to developing thymocytes. This diversity arises from both promiscuous gene expression and specialised differentiation programmes, including thymic mimetic‐cell populations, and is largely shaped by *AIRE*/*Aire* and *FEZF2*/*Fezf2*. Although the roles of *Aire* and *Fezf2* in regulating peripheral tissue antigen transcription are well established, a critical conceptual gap remains regarding how transcriptional promiscuity is translated into the repertoire of peptides presented by major histocompatibility complex class II (MHC‐II) molecules. In this review and perspective, we synthesize knowledge on mTEC biology, *Aire*‐ and *Fezf2*‐dependent transcriptional programmes, and advances in immunopeptidomics. We discuss regulatory layers that decouple mRNA abundance from peptide presentation and highlight how single‐cell transcriptomics and mass spectrometry–based immunopeptidomics provide complementary insights into mTEC heterogeneity and antigenic output. We argue that integrating these approaches is essential to understand central tolerance mechanistically and to explain selective defects in thymic self‐antigen presentation. This framework offers a refined basis for interpreting autoimmune disease origins and guiding antigen‐focused therapeutic strategies.

## Graphical abstract

Central tolerance is shaped not merely by promiscuous gene expression in medullary thymic epithelial cells (mTECs), but by the subset of self‐peptides ultimately presented on MHC class II molecules. We propose a multilayered framework in which AIRE/Aire and FEZF2/Fezf2, together with specialised differentiation programmes—including thymic mimetic‐cell populations—generate transcriptional diversity that is progressively filtered by posttranscriptional regulation, antigen processing, peptide editing and MHC‐II loading to produce the mTEC immunopeptidome. The resulting peptide repertoire is read by developing thymocytes through TCR signalling, thereby directing clonal deletion, regulatory T‐cell induction or survival of conventional T cells, and ultimately establishing central tolerance.

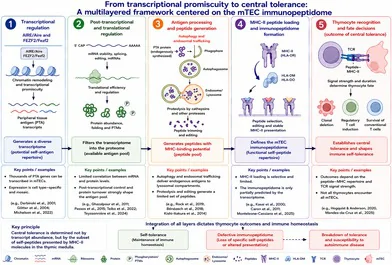

## 1 Central Immune Tolerance as a Multilayered Biological Process

The generation of a self‐tolerant T‐cell repertoire represents one of the most intricate and tightly regulated developmental programmes in vertebrate biology. This process must reconcile two opposing requirements: the need to generate a diverse pool of T lymphocytes capable of recognising foreign antigens and the imperative to prevent immune responses against self‐components. Central immune tolerance, established primarily in the thymus, is the basis of this balance and is essential for lifelong immune homeostasis [1, 2].

During thymic development, immature T lymphocytes (thymocytes) undergo a highly ordered sequence of differentiation and selection events. Positive selection in the thymic cortex ensures that thymocytes expressing T‐cell receptors (TCRs) capable of interacting with self–major histocompatibility complex (MHC) molecules receive survival signals. This step enforces MHC restriction and allows thymocytes to progress along the CD4^+^ or CD8^+^ lineage. Thymocytes that fail to recognise self‐MHC molecules undergo apoptosis and are eliminated [3, 4, 5, 6].

Following positive selection, thymocytes migrate to the thymic medulla, where they are exposed to a different selection pressure. Negative selection eliminates thymocytes bearing TCRs with high affinity for self‐antigens, preventing the emergence of strongly autoreactive T cells. In parallel, thymocytes receiving intermediate‐strength signals may be diverted into the regulatory T cell (Treg) lineage, contributing to peripheral tolerance. Thus, the medullary phase is not merely a deletion checkpoint but a critical decision node shaping both conventional and regulatory T‐cell compartments [3, 7, 8].

Medullary thymic epithelial cells (mTECs) are indispensable for these processes. Unlike most somatic cells, which express limited tissue‐specific gene sets, mTECs represent the molecular identity of the organism within a single niche. Through ectopic expression of thousands of peripheral tissue antigens (PTAs), mTECs recreate a molecular mosaic of self‐including proteins normally restricted to endocrine glands, exocrine tissues, nervous system, muscle, liver and skin. By presenting peptides derived from these antigens to developing thymocytes, mTECs provide a comprehensive self‐antigenic landscape that underpins central tolerance [9, 10, 11, 12].

The biological relevance of this system becomes evident when it fails. Defects in mTEC differentiation, antigen expression or presentation allow autoreactive T cells to escape into the periphery, leading to multiorgan autoimmunity. Both experimental models and human genetic disorders demonstrate that even subtle perturbations in thymic antigen presentation can have profound pathological consequences [13, 14, 15, 16].

Although the importance of mTECs in central tolerance has long been recognised, the conceptual focus of the field has evolved. Early models emphasized antigen expression as the defining feature of mTEC function, assuming that transcription of self‐antigens was sufficient to ensure immunological relevance [17, 18, 19, 20].

Nevertheless, evidence supports the idea that antigen expression alone does not equate to antigen presentation. Instead, the decisive functional endpoint is the immunopeptidome—the collection of peptides processed, loaded onto class I or class II MHC molecules and displayed at the cell surface [21, 22, 23, 24, 25, 26, 27, 28, 29, 30, 31, 32, 33].

In this context, the immunopeptidome emerges as the most proximal determinant of thymocyte fate. Thymocytes do not ‘see’ transcripts or proteins; they interrogate peptide–MHC complexes through their TCRs [34]. Consequently, understanding central tolerance requires moving beyond transcript‐centric descriptions toward an integrated view encompassing transcription, translation, antigen processing and peptide presentation. This multilayered perspective provides the framework for re‐evaluating how mTECs enforce self‐tolerance and sets the stage for exploring mechanisms connecting gene regulation to immune selection [22, 35].

## 2 Promiscuous Gene Expression (PGE): Scope, Organisation and Biological Meaning

PGE represents the most distinctive molecular hallmark of mTECs and is central to their role in immune tolerance. PGE refers to the ectopic transcription of thousands of genes that normally display highly restricted expression in peripheral tissues. These include genes characteristic of endocrine organs such as the pancreas and adrenal glands, exocrine tissues, liver, skeletal and cardiac muscle, skin and components of the nervous system. Through PGE, mTECs collectively recapitulate a molecular portrait of peripheral tissues within the thymic medulla [9, 36, 37, 38, 39]; (Matsumoto et al. 2025).

The scope of PGE is remarkable. Transcriptomic analyses have consistently shown that a substantial fraction of the genome can be detected in mTECs, including genes otherwise silent outside their tissue of origin. Importantly, this phenomenon is largely confined to the medullary epithelium and is not observed in cortical thymic epithelial cells or most other somatic cell types, underscoring its specialised and conserved nature (Derbinski et al. 2005); [9, 40].

Crucially, PGE is not the result of indiscriminate transcription. Multiple lines of evidence demonstrate that it is a regulated and developmentally programmed process that correlates with mTEC differentiation and maturation. As mTECs progress through developmental stages, they acquire the capacity to express increasing numbers and classes of tissue‐restricted antigens. This maturation‐dependent regulation indicates that PGE is embedded within broader transcriptional and epigenetic programmes defining mTEC identity [41, 42].

Recent single‐cell transcriptomic studies have refined the classical view of PGE by identifying specialised thymic epithelial populations known as mimetic cells. These cells do not merely express isolated PTA transcripts in a stochastic manner; rather, they activate coordinated transcriptional programmes that resemble those of specific peripheral cell types. Mimetic populations display molecular features of tuft cells, ciliated cells, enterocyte‐like cells, myoid cells and other tissue‐restricted lineages within the thymic medulla, revealing an unexpected level of epithelial specialisation [18, 43, 44, 45]. This finding indicates that at least part of the antigenic diversity generated within the thymus arises through regulated cellular differentiation programmes rather than through purely random transcriptional events. Importantly, single cell analyses also demonstrate that individual mTECs express only a limited subset of PTA transcripts at any given time, indicating that broad self‐antigen representation emerges from the combined contribution of specialised mimetic populations and heterogeneous PTA expression across the mTEC compartment.

This mosaic architecture has important functional implications. By distributing PTA expression across many mTECs, the thymus minimises metabolic burden on individual cells while maximising antigen diversity presented to thymocytes. Such heterogeneity likely increases the probability that thymocytes migrating through the medulla encounter relevant self‐antigens, enhancing negative selection and regulatory T cell induction [46, 47, 48].

Historically, the biological meaning of PGE has been interpreted through a transcript‐centric lens. The prevailing assumption was that transcription of PTA‐encoding mRNAs in mTECs was sufficient to ensure immunological representation of those self‐antigens. In this view, detection of a transcript implied that the corresponding protein—and its peptides—would be available for presentation to developing thymocytes.

However, this assumption oversimplifies gene expression and antigen presentation. Between transcription and thymocyte recognition lie multiple regulatory layers, including posttranscriptional control of mRNA stability, translational efficiency, protein folding and turnover, intracellular trafficking and selective routing into antigen‐processing pathways. Only a fraction of cellular proteins is processed into peptides capable of binding MHC class II molecules, and competition among peptides further constrains which antigens reach the cell surface [49, 50].

In mTECs, these regulatory layers are likely especially consequential. PGE generates many low‐abundance or transient transcripts, raising the possibility that a substantial proportion of PTA mRNAs may never be translated into stable proteins or incorporated into the MHC‐II–associated peptidome. As a result, the transcriptional landscape defined by PGE may overestimate the antigenic information functionally presented to thymocytes.

Recognising this disconnect compels a re‐evaluation of the biological meaning of PGE. Rather than viewing promiscuous transcription as a direct proxy for antigen presentation, PGE should be understood as the upstream substrate upon which multiple filtering steps shape the final immunopeptidome.

## 3 Aire: A Noncanonical Regulator of Transcriptional Promiscuity

The autoimmune regulator (Aire) protein is the most extensively characterised molecular driver of PGE in TECs and has become a paradigm for unconventional transcriptional regulation. Unlike classical transcription factors, which recognise specific DNA motifs and directly activate promoters, Aire protein functions primarily as a transcriptional facilitator. Its activity is directed toward genes embedded in repressive chromatin environments, many of which are otherwise transcriptionally silent outside their tissue of origin [37, 38, 51, 52, 53]. The pivotal role of the *Aire* gene in central tolerance was established by Anderson and colleagues, who demonstrated that *Aire* deficiency leads to reduced thymic expression of a broad set of PTAs and impaired deletion of autoreactive T cells [54]. This landmark study provided the first direct evidence that ectopic expression of tissue‐restricted antigens within the thymus is a critical mechanism for establishing self‐tolerance and preventing organ‐specific autoimmunity. These findings transformed the understanding of central tolerance by linking *Aire*‐dependent transcriptional regulation to thymocyte selection and autoimmune disease susceptibility.

Mechanistically, Aire protein does not act as a simple on–off switch. Instead, it interacts with multiple components of the transcriptional and chromatin‐remodelling machinery to promote transcriptional permissiveness. Aire protein associates with RNA polymerase II complexes, facilitating release of stalled polymerase at promoter‐proximal regions [52, 55]. Aire protein also interacts with chromatin‐associated proteins and recognises histone modifications characteristic of inactive or poised chromatin states, such as unmethylated H3K4. Through these interactions, Aire protein enables transcriptional elongation at loci otherwise refractory to expression, expanding the transcriptional repertoire of mTECs [38, 56].

This noncanonical mode explains several defining features of *Aire*‐dependent gene regulation. *Aire*‐controlled genes are often expressed at low levels, display heterogeneous transcription and lack the coordinated promoter architecture typical of tissue‐specific gene expression [57, 58].

The clinical significance of *AIRE* became evident with the identification of loss‐of‐function mutations in this gene as the cause of autoimmune polyendocrinopathy‐candidiasis‐ectodermal dystrophy (APECED), also known as autoimmune polyendocrine syndrome type 1 (APS‐1) (https://omim.org/entry/240300). Two independent studies published in 1997 established *AIRE* as the disease‐causing gene, providing the first direct genetic evidence that defects in thymic self‐tolerance can result in multiorgan autoimmunity in humans [59, 60]. These discoveries laid the foundation for subsequent investigations into the molecular mechanisms by which *AIRE*/*Aire* regulates central tolerance [61].

Murine models of *Aire* deficiency have provided complementary insights. *Aire*‐deficient mice develop multiorgan autoimmunity and exhibit defects in negative selection within the thymus. At the cellular level, Aire loss alters mTEC differentiation trajectories, reduces expression of tissue‐restricted antigens and disrupts thymocyte deletion efficiency. These defects extend beyond antigen expression to broader aspects of thymic architecture and cell–cell interactions [37, 53, 62].

One of the most significant advances has been the recognition that *Aire*‐dependent transcription extends beyond PTAs. Transcriptomic analyses show that *Aire* regulates genes involved in cell adhesion, extracellular matrix organisation, cytokine and chemokine signalling and intracellular trafficking. For example, *Aire* influences adhesion molecules that stabilise interactions between mTECs and developing thymocytes, a prerequisite for effective antigen presentation and signal integration during negative selection [38, 63, 64, 65]; (Huoh et al. 2020). *Aire* contributes to mTEC morphology, as different mutations in this gene influence cellular shape [66]. However, *Aire* deficiency does not impair mTEC differentiation into the autoantigen‐presenting stage [67].

In addition to protein‐coding genes, *Aire* controls noncoding RNAs including miRNAs and long noncoding RNAs. These regulatory RNAs introduce additional layers of posttranscriptional control that modulate mTEC function, thymocyte interactions and possibly antigen‐processing pathways. Such findings reinforce the view of *Aire* as a pleiotropic regulator acting at multiple regulatory levels within mTECs [68, 69, 70].

This pleiotropy has important implications for *Aire*‐dependent tolerance mechanisms. Rather than acting solely as a switch for PTA expression, *Aire* shapes a permissive molecular environment in which transcriptional diversity, cellular interactions and regulatory RNA networks converge to support central tolerance. Consequently, the effects of *Aire* deficiency cannot be explained solely by the loss of individual self‐antigens.

From the perspective of antigen presentation, this broader role of Aire raises critical questions. Although *Aire*‐driven transcription expands the pool of potential antigens, it does not directly dictate which proteins are translated, processed and presented as peptides by MHC‐II molecules. Many *Aire*‐induced transcripts may never contribute to the immunopeptidome, whereas others may disproportionately influence thymocyte selection despite modest transcriptional levels. Thus, *Aire*'s ultimate impact on immune tolerance is realised not at transcription but at the downstream interface where gene expression is filtered through translational and antigen‐processing pathways.

Recognising *Aire* as a noncanonical and pleiotropic regulator reinforces a central theme of this review: transcriptional control, although necessary and fundamental for understanding mTEC molecular genetics, is insufficient to predict functional antigen presentation. Understanding how *Aire*‐dependent transcription is translated into the mTEC immunopeptidome is essential for a mechanistic interpretation of central tolerance and its failure in autoimmune disease.

## 4 *Fezf2*: A Complementary Axis of Autoantigen Regulation

The identification of FEZ family zinc finger 2 (*Fezf2*) as a second major regulator of PTA expression in mTECs represented a significant conceptual advance in central tolerance. Prior to this discovery, *Aire* was widely regarded as the dominant driver of PGE in mTECs. Recognition that the *Fezf2* gene controls a substantial and largely distinct subset of tissue‐restricted antigens altered this view, revealing that antigen regulation in the thymic medulla is more modular and multifaceted than previously appreciated [71, 72, 73].

In contrast to Aire, Fezf2 protein functions as a classical DNA‐binding transcription factor. Its zinc finger domains enable direct interaction with specific genomic loci, leading to conventional modes of transcriptional activation. This mechanistic distinction has important consequences for Fezf2‐dependent gene expression. Whereas Aire preferentially activates genes in repressive chromatin environments through facilitation of transcriptional elongation, Fezf2 protein promotes transcription through sequence‐specific binding and recruitment of transcriptional machinery [72, 73].

Transcriptomic analyses show that PTAs regulated by the *Fezf2* gene overlap only partially with the *Aire*‐dependent repertoire. A substantial fraction of tissue‐restricted genes expressed in mTECs is controlled exclusively by *Fezf2*, while others require both regulators. This partitioning suggests that distinct classes of self‐antigens are governed by different regulatory logics, possibly reflecting differences in genomic organisation, chromatin context or evolutionary origin [72, 73, 74].

The functional relevance of *Fezf2*‐dependent regulation has been established through genetic studies. *Fezf2*‐deficient mice develop spontaneous autoimmune phenotypes and exhibit defects in negative selection comparable in some respects to those observed in *Aire*‐deficient animals. Importantly, these manifestations arise despite intact *Aire* function, demonstrating that *Aire*‐independent pathways are indispensable for central tolerance. Together, these findings indicate that *Fezf2* is a core component of thymic tolerance mechanisms [75].

The existence of *Fezf2*‐dependent PTAs challenges earlier models placing *Aire* at the centre of transcriptional promiscuity in mTECs. Instead, the emerging picture is a distributed regulatory architecture in which multiple transcriptional programmes operate in parallel to generate antigenic diversity. Such modular organisation may confer robustness, ensuring that failure of a single pathway does not completely abrogate self‐antigen representation in the thymus.

Beyond PTAs, *Fezf2* may influence broader aspects of mTEC biology. Although less extensively characterised than *Aire*, evidence suggests that *Fezf2*‐regulated genes include factors involved in metabolism, differentiation and possibly antigen‐processing pathways. Given *Fezf2*'s established roles in neuronal development, where it governs cell fate decisions and connectivity, it is plausible that *Fezf2* exerts similar pleiotropic effects in mTECs, shaping antigen expression and cellular states relevant to thymocyte interaction [76].

From a conceptual standpoint, the coexistence of *Aire*‐ and *Fezf2*‐dependent transcriptional programmes raises important questions about their integration. Do these regulators act in distinct mTEC subsets or maturation stages, or converge within the same cells to produce combinatorial antigenic outputs? How are their activities coordinated temporally and epigenetically during mTEC differentiation? And critically, how do differences in transcriptional regulation translate into differences in antigen processing and presentation?

Addressing these questions requires moving beyond transcriptomic descriptions toward functional readouts. Although *Aire* and *Fezf2* shape the pool of potential self‐antigens at the mRNA level, it remains uncertain how their programmes are filtered through translational control, proteolytic processing and MHC‐II loading. The degree to which Aire‐ and Fezf2‐dependent transcripts are represented in the mTEC immunopeptidome—and whether they contribute differentially to thymocyte deletion or regulatory T cell induction—remains a central unresolved issue.

## 5 The Transcriptome–Proteome–Peptidome Disconnect

One of the most persistent misconceptions in both immunology and molecular biology is the assumption of a linear and proportional relationship between mRNA abundance, protein expression and peptide presentation. In this simplified view, transcriptional output is treated as a reliable proxy for cellular function. However, quantitative analyses across diverse biological systems have shown that correlations between transcript levels and protein abundance are, at best, moderate [77, 78, 79].

When the endpoint is peptide presentation by MHC molecules, this correlation may be even weaker. Several large‐scale multiomics studies have demonstrated that mRNA abundance is only a partial predictor of protein abundance. Across diverse biological systems, transcript levels typically explain between 30% and 60% of the observed variance in protein expression, indicating the existence of extensive regulatory control beyond transcription [77, 78, 80]; (Payne et al. 2015). Mechanisms such as alternative splicing, differential mRNA stability, translational efficiency, protein folding, posttranslational modification and protein turnover collectively contribute to this disconnect. Consequently, the functional proteome represents a highly filtered version of the transcriptome rather than a direct reflection of it.

Large‐scale proteomic and transcriptomic studies have shown that mRNA abundance explains only a fraction of the variance observed at the protein level. This disconnect arises from multiple regulatory layers between transcription and translation, including mRNA processing, nuclear export, stability and translational efficiency. Protein abundance is further shaped by folding efficiency, posttranslational modifications, subcellular localisation and degradation rates (Payne et al. 2015) [80]. As a result, protein levels cannot be reliably inferred from transcriptomic data alone.

The disconnect becomes even more pronounced when considering self‐antigen presentation. Only a subset of cellular proteins gains access to antigen‐processing pathways, and only a fraction of processed peptides is loaded onto MHC molecules and displayed at the cell surface. Peptide presentation is constrained by multiple selective steps, including proteolytic cleavage specificity, peptide stability, competition for MHC binding and chaperone‐mediated editing within endosomal compartments.

A critical component of this editing process is the coordinated action of MHC‐II–associated chaperones and peptide editors. Newly synthesised MHC‐II molecules initially associate with the invariant chain (CD74), which stabilises the complex and prevents premature peptide loading during intracellular trafficking. Within endosomal compartments, CD74 is progressively degraded, leaving the class II‐associated invariant chain peptide (CLIP) bound to the peptide‐binding groove. H2‐DM (HLA‐DM in humans) catalyzes the release of CLIP and promotes the exchange of low‐affinity peptides for more stable peptide–MHC‐II complexes. In contrast, H2‐O (HLA‐DO) modulates H2‐DM activity and contributes to controlling the quality and diversity of the MHC‐II immunopeptidome. Through this peptide‐editing system, only a subset of available peptides achieves stable surface presentation, introducing an additional layer of selectivity between protein expression and thymocyte recognition [81, 82]; (Margulies et al. 2022).

An additional source of selectivity arises from competition among peptides for occupancy of MHC‐II binding grooves. Because the number of available MHC‐II molecules is limited, peptides generated from different proteins must compete for stable binding and surface display. Peptides with higher affinity, greater kinetic stability or more favorable access to antigen‐processing compartments are preferentially retained, whereas many low‐abundance or weakly binding peptides fail to reach detectable levels at the cell surface. Consequently, the representation of a given antigen within the immunopeptidome depends not only on its expression level but also on its competitive ability within the peptide‐loading environment. This competitive selection further contributes to the disconnect between transcript abundance and peptide presentation [83, 84].

Thus, the relationship between mRNA expression and MHC‐bound peptides is highly nonlinear and subject to stringent filtering.

Antigen presentation imposes an additional level of biological selection beyond protein expression. Proteins differ substantially in their accessibility to endosomal and lysosomal processing pathways, their susceptibility to proteolytic cleavage and their capacity to generate peptides compatible with MHC‐II binding grooves. As a result, only a subset of cellular proteins yields peptides that successfully compete for stable MHC‐II presentation. Consequently, only a fraction of cellular proteins contributes measurably to the immunopeptidome, and peptide abundance frequently diverges from both transcript and protein abundance [49, 85].

In mTECs, these regulatory layers are likely amplified by the unusual nature of PGE. PGE generates a transcriptional landscape characterised by low‐level, stochastic and often transient expression of tissue‐restricted genes [86]. Many PTA transcripts are expressed at levels orders of magnitude lower than in their native tissues, raising questions about their translational competence and biological relevance. Moreover, some transcripts generated through PGE may be incomplete, alternatively spliced or otherwise atypical, further limiting their contribution to the proteome. These considerations may be particularly relevant in mTECs, where PGE often produces low‐abundance and transient transcripts. Because many PTA‐encoding transcripts are expressed at levels considerably lower than those observed in their native peripheral tissues, their translation into stable proteins and subsequent incorporation into antigen‐processing pathways cannot be assumed. Thus, transcriptional promiscuity generates a broad repertoire of potential self‐antigens, whereas thymocyte selection is ultimately determined by the smaller subset of peptides that successfully reaches the MHC‐II immunopeptidome.

Consequently, the presence of a PTA‐encoding mRNA in mTECs does not guarantee that the corresponding protein will be synthesized in sufficient quantity—or at all. Even when translation occurs, there is no assurance that the protein will be routed into endosomal or lysosomal pathways required for MHC‐II processing. Cytosolic or nuclear proteins, for example, may be preferentially degraded through proteasomal pathways without contributing significantly to the MHC‐II–associated peptidome.

An additional layer of complexity arises from competition within the antigen‐processing machinery. Peptides derived from abundant housekeeping proteins can dominate the MHC‐II binding landscape, potentially excluding low‐abundance PTA‐derived peptides. Chaperones such as CD74 and peptide editors such as H2‐DM and H2‐O further shape the peptide repertoire by favouring stable peptide–MHC complexes [85]. Cytokine‐mediated cues, particularly interferon‐γ, can dynamically modulate MHC‐II expression and antigen‐processing capacity, introducing context‐dependent variability into the immunopeptidome [50].

Taken together, these considerations underscore a fundamental conceptual shift required in studies of central tolerance. Transcriptomic analyses, while invaluable for identifying potential antigenic sources, cannot predict which self‐antigens are functionally presented to developing thymocytes. Thymocytes do not respond to mRNA abundance or protein expression per se; they respond to peptide–MHC complexes encountered during migration through the thymic medulla [87]. These peptide–MHC interactions are interpreted through TCR signalling thresholds that ultimately determine thymocyte fate, including clonal deletion, regulatory T‐cell differentiation or survival as conventional T cells.

This realisation necessitates a transition from transcriptomic inference to direct interrogation of peptide presentation. Immunopeptidomics, by providing an unbiased snapshot of peptides bound to MHC molecules, offers a functional readout bridging gene regulation and immune selection. In mTECs, such approaches are essential to determine which *Aire*‐ or *Fezf2*‐dependent transcripts are translated into immunologically relevant signals.

By reframing central tolerance around the immunopeptidome rather than the transcriptome, this perspective challenges long‐standing assumptions and opens new avenues for understanding how failures in antigen processing or presentation—rather than gene expression alone—may contribute to autoimmunity. In this sense, the transcriptome–proteome–peptidome disconnect is not merely a technical limitation, but a biological feature that adds robustness and selectivity to self‐tolerance.

## 6 Immunopeptidomics: Direct Access to the Functional Antigenic Repertoire

Immunopeptidomics, based on high‐resolution mass spectrometry, has emerged as a powerful and unbiased strategy to characterise the repertoire of peptides bound to MHC molecules and it has been used for several years now [26, 88, 89]. Unlike transcriptomic or proteomic approaches, which infer potential antigenic sources, immunopeptidomics provides direct access to peptides displayed at the cell surface and interrogated by TCRs. In mTECs, this distinction is particularly consequential, as thymocyte fate is dictated exclusively by peptide–MHC complexes rather than upstream molecular intermediates.

Applied to mTECs, immunopeptidomics enables direct interrogation of the self‐peptides that define the antigenic landscape of the thymic medulla. This approach allows investigators to move beyond theoretical models of antigen expression and address a fundamental question of central tolerance: which self‐antigens are truly represented at the level of peptide presentation? By identifying MHC‐bound peptides, immunopeptidomics captures the integrated output of transcription, translation, protein turnover and antigen‐processing pathways, offering a functional readout of mTEC activity.

Although direct immunopeptidomic analyses of thymic epithelial cells remain relatively scarce, early studies already suggested that peptide presentation differs substantially between TEC subsets. Kasai et al. [90] demonstrated that CLIP‐derived peptides bound to MHC‐II molecules in mTECs differed from those found in cortical thymic epithelial cells with respect to peptide diversity, length and C‐terminal processing. These findings provided early evidence that antigen presentation in the thymus is shaped by cell type‐specific processing mechanisms and highlighted the importance of examining peptide repertoires directly rather than inferring them solely from transcriptional profiles.

Recent advances in mass spectrometry instrumentation have expanded the feasibility of immunopeptidomic studies [88]. High‐resolution platforms enable detection of thousands of MHC‐bound peptides from limited starting material. Improvements in chromatographic separation, ionisation efficiency and acquisition strategies have increased depth and reproducibility, while bioinformatics pipelines tailored to immunopeptidomic data facilitate confident peptide identification and annotation. Together, these developments have made it possible to apply immunopeptidomics to previously inaccessible systems, including thymic epithelial subsets.

Beyond its technical advantages, immunopeptidomics represents a conceptual shift in the study of central tolerance. Transcriptomic analyses define the pool of genes that may contribute to self‐antigen expression and therefore reflect the potential antigenic capacity of mTECs. In contrast, immunopeptidomics identifies the peptides that are processed, loaded onto MHC molecules and displayed to developing thymocytes, thereby revealing the realised antigenic output of these cells.

As proposed by Caron et al. [91], the immunopeptidome constitutes an integrated readout of cellular regulation, reflecting the combined effects of transcription, translation, protein turnover and antigen processing. This distinction is particularly important in the thymus because thymocyte fate is determined exclusively by peptide–MHC recognition rather than by transcript or protein abundance. Consequently, while transcriptomics reveals what mTECs can potentially present, immunopeptidomics reveals what they effectively present, making it the most functionally relevant layer for understanding central tolerance. This distinction is crucial for understanding thymocyte selection, as only presented peptides can contribute to negative selection or regulatory T‐cell induction.

In the context of *Aire*‐ and *Fezf2*‐dependent regulation, immunopeptidomics provides a powerful means to directly test long‐standing assumptions. It allows direct assessment of whether transcriptionally induced PTAs are represented in the MHC‐II–associated peptidome and whether differences in transcriptional control translate into qualitative or quantitative differences in peptide presentation.

Importantly, immunopeptidomics also enables comparative analyses across genetic or environmental perturbations. By directly comparing peptide repertoires of wild‐type, *Aire*‐ or *Fezf2*‐deficient mTECs, it becomes possible to delineate how regulatory pathways shape antigenic signals encountered by developing thymocytes. Such comparisons provide mechanistic insight into how alterations in peptide presentation—rather than transcription alone—may trigger defects in central tolerance and predisposition to autoimmunity.

Despite recent technological advances, direct characterisation of the mTEC immunopeptidome remains comparatively unknown relative to transcriptomic studies. As a result, our understanding of how *Aire*‐ and *Fezf2*‐dependent transcriptional programmes are translated into peptide repertoires is still incomplete. Expanding immunopeptidomic investigations of defined thymic epithelial populations represents an important priority for the field and will be essential for establishing a mechanistic link between gene regulation, antigen presentation and thymocyte selection.

## 7 Single‐Cell Transcriptomics and mTEC Heterogeneity

The advent of single‐cell RNA sequencing (scRNA‐seq) has profoundly reshaped our understanding of mTEC biology. What was once considered a relatively uniform population is now recognised as a heterogeneous compartment composed of multiple subsets differing in developmental stage, functional specialisation and transcriptional programme [69, 92, 93]. This diversity has important implications for central tolerance, suggesting that antigen expression and presentation are distributed unevenly across the mTEC population.

Crucially, these subsets differ not only in their repertoire of expressed PTAs but also in genes involved in antigen processing and presentation. Variations in expression of proteases, peptide editors, chaperones and MHC‐II molecules imply that the efficiency and qualitative nature of peptide presentation may vary substantially between subsets. As a result, the immunological impact of a given PTA transcript likely depends on the cellular context in which it is expressed [69].

As introduced above (Section 2), an additional dimension of mTEC heterogeneity has emerged from the identification of thymic mimetic cells, specialised epithelial populations that recapitulate transcriptional programmes characteristic of peripheral tissues. Single‐cell transcriptomic analyses revealed subsets resembling tuft cells, enterocyte‐like cells, ciliated epithelial cells, myoid cells and other tissue‐restricted lineages, each expressing lineage‐defining transcription factors and coordinated gene modules rather than isolated PTAs [18, 44].

Importantly, these mimetic‐cell programmes are associated with the expression of lineage‐defining transcription factors, including *Pou2f3* in tuft‐like cells, *Hnf4g* in enterocyte‐like cells, *Foxj1* in ciliated epithelial cells and *Myog* in myoid cells, which promote tissue‐specific chromatin landscapes and coordinated transcriptional modules characteristic of their respective peripheral counterparts [44]. These findings indicate that self‐antigen representation in the thymus is achieved not only through stochastic PGE but also through regulated differentiation programmes that generate functionally distinct epithelial states.

The existence of mimetic cells further reinforces the concept that antigen presentation is highly context dependent. Because these populations differ in transcriptional identity, differentiation status and expression of antigen‐processing machinery, they may contribute unequally to the thymic immunopeptidome. Consequently, the presence of a tissue‐restricted transcript within a given mimetic population does not necessarily predict its representation at the peptide level. Integrating single‐cell transcriptomics with immunopeptidomics will therefore be essential to determine how individual mimetic‐cell populations contribute to the repertoire of self‐peptides that shape thymocyte selection and central tolerance.

This view of mTEC heterogeneity has important implications for central tolerance. Rather than being enforced uniformly by all mTECs, tolerance may arise from the coordinated action of specialised subsets, each contributing distinct antigenic and signalling inputs. Thymocytes migrating through the medulla therefore encounter a dynamic and spatially heterogeneous antigenic landscape shaped by the distribution and functional properties of mTEC subsets [87].

Integrating scRNA‐seq data with immunopeptidomic profiles offers a powerful strategy to link cellular heterogeneity with functional antigen presentation. Such approaches address questions that cannot be resolved by either technology alone. For example, do specific mTEC subsets disproportionately contribute to the MHC‐II–associated peptidome? Are *Aire*‐ or *Fezf2*‐dependent transcripts preferentially translated and presented in cellular states? And how does disruption of these regulatory pathways reshape antigenic output across mTEC populations?

By mapping immunopeptidomic data onto single‐cell–defined subsets, it becomes possible to assign functional antigen presentation to specific mTEC states. This approach can reveal whether certain subsets act as dominant sources of self‐peptides or whether antigenic diversity emerges from cumulative contributions across the population. Comparative analyses in *Aire*‐ or *Fezf2*‐deficient contexts can elucidate how genetic perturbations selectively affect mTEC subsets rather than uniformly altering the compartment.

Such integration also has implications for understanding disease mechanisms. If autoimmune phenotypes arise from selective loss or alteration of self‐antigen presentation in specific mTEC subsets, this could explain the tissue specificity and variability observed in many autoimmune disorders. Single‐cell–resolved immunopeptidomics therefore holds promise not only for refining models of central tolerance but also for identifying subset‐specific vulnerabilities underlying immune dysregulation.

## 8 Implications for Thymocyte Selection and Autoimmunity

The qualitative and quantitative composition of the mTEC immunopeptidome exerts a decisive influence on thymocyte fate decisions. Developing thymocytes interrogate the thymic environment exclusively through interactions between their TCRs and self‐peptide–MHC complexes displayed on antigen‐presenting cells [46, 87]. The strength, duration and context of these interactions determine whether a thymocyte undergoes clonal deletion, differentiates into a regulatory T cell or survives to enter the peripheral T‐cell pool.

Strong TCR engagement by self‐peptide–MHC complexes typically induces apoptotic deletion, eliminating thymocytes with high autoreactive potential. In contrast, intermediate‐strength signals can divert thymocytes toward the thymic regulatory T cell (tTreg) lineage, contributing to peripheral tolerance. Weak interactions are generally permissive and allow thymocytes to survive as conventional T cells. Thus, thymocyte selection is not binary but a graded response to quantitative and qualitative features of antigen presentation [94, 95, 96].

Although thymocyte selection is highly sensitive to peptide–MHC interactions, the extent to which quantitative alterations in peptide presentation alone is sufficient to cause autoimmune disease remains incompletely understood. Current evidence strongly supports the role for antigen presentation in shaping T‐cell repertoires, but the pathological consequences of immunopeptidomic variation are likely influenced by additional factors, including MHC genotype, TCR repertoire composition, thymic microenvironment and peripheral tolerance mechanisms. Consequently, alterations in peptide presentation should be viewed as one component of a broader regulatory network governing immune self‐tolerance.

Within this framework, alterations in the immunopeptidome have the potential to influence thymocyte fate decisions by modifying the peptide–MHC signals encountered during thymic selection. Changes in peptide abundance, stability or presentation kinetics could shift TCR signalling thresholds and thereby affect the balance between deletion, regulatory T‐cell induction and survival. However, the extent to which relatively subtle immunopeptidomic changes translate into clinically relevant autoimmune phenotypes remains an important unresolved question.

Defects in the mTEC immunopeptidome may arise through multiple mechanisms. Loss of regulatory pathways such as *Aire* or *Fezf2* can reduce or eliminate presentation of distinct subsets of self‐peptides. Importantly, defects in central tolerance may arise even when transcriptional programmes remain largely intact. Alterations in antigen processing, peptide editing or MHC‐II loading can selectively modify the representation of individual self‐peptides within the immunopeptidome. Such changes may alter thymocyte selection thresholds and create antigen‐specific vulnerabilities without requiring global disruption of gene expression. This framework may help explain why autoimmune diseases often target specific tissues despite relatively subtle molecular defects in thymic tolerance mechanisms.

Evidence from studies of *Aire* deficiency, antigen‐processing defects and MHC‐associated peptide editing supports the concept that alterations in peptide presentation can influence the efficiency of central tolerance. Nevertheless, most available data demonstrate changes in thymocyte selection indirectly, and direct causal links between specific quantitative changes in thymic peptide repertoires and autoimmune disease development remain limited [54, 97]. Future studies combining immunopeptidomics with functional analyses of thymocyte selection will be required to establish these relationships more rigorously.

Alternatively, changes in antigen‐processing efficiency, peptide editing or MHC‐II expression may reshape the peptide repertoire without altering transcriptional programmes. In either case, immunopeptidomic alterations directly affect the signals perceived by developing thymocytes.

This perspective provides a plausible framework for understanding the organ specificity observed in many autoimmune disorders. *Aire*‐ or *Fezf2*‐related autoimmunity often targets restricted tissues rather than producing uniform systemic disease. Such selectivity may reflect loss or underrepresentation of specific tissue‐derived peptides in the thymic immunopeptidome, leading to incomplete deletion of autoreactive T cells. In this model, autoimmunity emerges not from a global failure of tolerance, but from selective ‘holes’ in the antigenic repertoire presented during thymocyte development.

Moreover, alterations in peptide abundance may influence not only clonal deletion but also regulatory T cell generation. Reduced presentation of certain self‐peptides could impair induction of tTregs with specific antigenic specificities, weakening peripheral tolerance. Conversely, changes in peptide presentation dynamics may favour regulatory outcomes for some antigens while permitting escape of autoreactive clones for others, further contributing to disease heterogeneity.

At present, much of the evidence linking thymic immunopeptidome composition to autoimmune susceptibility remains inferential. Nevertheless, the growing recognition that thymocytes respond exclusively to peptide–MHC complexes provides a strong rationale for investigating the immunopeptidome as a biologically relevant layer connecting gene regulation, antigen presentation and immune tolerance.

Integration of immunopeptidomic data with functional studies of thymocyte selection offers an opportunity to dissect these mechanisms in detail. By correlating changes in the peptide repertoire with thymocyte fate, it becomes possible to identify which self‐peptides are critical for tissue tolerance. Such insights may also help explain interindividual variability in autoimmune susceptibility, even in the presence of shared genetic risk factors.

## 9 Conceptual Framework: From PGE to Thymocyte Fate

### 9.1 Central Premise

We propose a hierarchical framework linking PGE to central tolerance through five sequential and functionally distinct layers. The first four layers describe biological processes that progressively filter transcriptional diversity into a functional immunopeptidome, whereas the fifth layer represents the biological outcome of these processes at the level of thymocyte selection and central tolerance (see the Graphical Abstract). This organisation emphasizes that transcript abundance alone is insufficient to predict tolerance outcomes because thymocytes ultimately respond to peptide–MHC complexes rather than transcripts or proteins.

#### 9.1.1 Layer 1—Transcriptional Regulation of PGE

*Aire* and *Fezf2* drive partially overlapping transcriptional programmes that induce stochastic expression of thousands of tissue‐restricted genes in mTECs. This generates extensive transcriptomic diversity across the mTEC population without ensuring functional antigen presentation.

#### 9.1.2 Layer 2—Transcriptome Filtering (Multilayer Antigen Processing)

Only a fraction of PTA mRNAs is efficiently translated. Posttranscriptional regulation, mRNA stability, translation efficiency and protein turnover create a major filter between gene expression and antigen availability.

#### 9.1.3 Layer 3—Antigen Processing (Immunopeptidome Layer)

Proteins entering endosomal–lysosomal pathways are selectively processed into peptides. Competition for MHC‐II loading, chaperone activity (e.g., CD74, H2‐DM/H2‐O) and inflammatory cues such as IFN‐γ further shape the peptide repertoire. The resulting immunopeptidome represents the true antigenic output of mTECs.

#### 9.1.4 Layer 4—Thymocyte Selection and Tolerance

TCR signalling and thymocyte fate decisions. Developing thymocytes interrogate the mTEC immunopeptidome through interactions between their TCRs and peptide–MHC complexes. High‐affinity interactions promote clonal deletion, intermediate signals support thymic regulatory T‐cell differentiation and weaker interactions permit survival of conventional T cells. Thus, the immunopeptidome generated by the preceding layers is translated into biologically meaningful signalling events that determine thymocyte fate.

#### 9.1.5 Layer 5—Determinant of Central Tolerance

The peptide–MHC repertoire generated through the preceding layers is interpreted by developing thymocytes during their interactions with mTECs. Depending on the strength and quality of TCR engagement, thymocytes undergo clonal deletion, differentiate into regulatory T cells or survive as conventional T cells. Thus, Layer 5 represents the functional outcome of peptide presentation and TCR signalling, culminating in the establishment of central tolerance.

### 9.2 Conceptual Implication

This framework shifts the focus of central tolerance research from transcriptional promiscuity alone to the sequence of biological processes that ultimately determine peptide presentation and thymocyte fate. Integrative analyses combining single‐cell transcriptomics with immunopeptidomics are therefore essential to understand how *Aire*‐ and *Fezf2*‐dependent transcriptional programmes are translated into functional antigen presentation and self‐tolerance.

Importantly, the fifth layer does not represent an additional regulatory mechanism acting upon the immunopeptidome. Rather, it represents the biological interpretation of the immunopeptidomic output generated by the upstream layers. In this view, central tolerance emerges from dynamic interactions between developing thymocytes and heterogeneous mTEC populations, providing a functional bridge between molecular regulation and immune homeostasis.

## 10 Concluding Remarks and Perspectives

Despite major advances in our understanding of *Aire*‐ and *Fezf2*‐dependent gene regulation, several fundamental questions remain unresolved. A central challenge is determining which fraction of transcriptionally expressed PTAs is ultimately translated, processed and incorporated into the MHC‐II immunopeptidome. This issue has gained additional complexity with the recent recognition of mTEC heterogeneity and thymic mimetic‐cell populations, which suggest that antigen presentation may be distributed unevenly across specialised epithelial subsets [44, 69]. At present, the relationship between transcriptomic diversity and functional peptide presentation remains incompletely understood.

*AIRE*/*Aire* and *FEZF2*/*Fezf2* shape central tolerance not only by driving PGE but by determining the repertoire of self‐peptides presented by mTECs. Integrating transcriptomic and immunopeptidomic data is essential to understand how gene expression translates into antigen presentation. This framework provides a mechanistic basis for explaining selective defects in central tolerance and the molecular origins of autoimmune disease.

Defining disease‐associated immunopeptidomes together with relevant MHC molecules and cognate TCR repertoires will be critical for understanding how self‐peptides escape thymic control and drive pathogenic immune responses. Such knowledge may guide antigen‐focused therapies, including peptide‐based strategies to modulate autoreactive T‐cell recognition, supporting more precise interventions in autoimmune disease.

## Funding

This work was funded by the following agencies, Coordenação de Aperfeiçoamento de Pessoal de Nível Superior (CAPES, Ministry of Education of Brazil, Brasília, Brazil throughout funding code 001), Fundação de Amparo à Pesquisa do Estado de São Paulo (FAPESP Grant # 2025/09892‐9 to G.A.P., V.M.F. and E.A.D., and 2023/17154‐2 to V.M.F., G.A.P., and A.P.M.), and Conselho Nacional de Desenvolvimento Científico e Tecnológico (CNPq, Brasília, Brazil Grant # 311304/2021‐4, and # 302828/2026‐5 to G.A.P., Grant # 316934/2021 to V.M.F. and Grant # 303782/2024‐2 to E.A.D.).

## Disclosure

This article was written by the authors. Artificial intelligence tools were used solely for English language editing, stylistic revision and the graphical abstract art.

## Conflicts of Interest

The authors declare no conflicts of interest.

## Data Availability

Data sharing not applicable to this article as no datasets were generated or analysed during the current study.
